# Activation of Local and Systemic Defence Responses by Flg22 Is Dependent on Daytime and Ethylene in Intact Tomato Plants

**DOI:** 10.3390/ijms22158354

**Published:** 2021-08-03

**Authors:** Zalán Czékus, András Kukri, Kamirán Áron Hamow, Gabriella Szalai, Irma Tari, Attila Ördög, Péter Poór

**Affiliations:** 1 Department of Plant Biology, Faculty of Science and Informatics, University of Szeged, 6726 Szeged, Hungary; czekus.z@bio.u-szeged.hu (Z.C.); kukri.andras2@gmail.com (A.K.); tari@bio.u-szeged.hu (I.T.); aodog@bio.u-szeged.hu (A.Ö.); 2 Doctoral School of Biology, University of Szeged, 6726 Szeged, Hungary; 3 Department of Plant Physiology, Agricultural Institute, Centre for Agricultural Research of the Hungarian Academy of Sciences, 2462 Martonvásár, Hungary; hamow.kamiran@atk.hu (K.Á.H.); szalai.gabriella@atk.hu (G.S.)

**Keywords:** dark, ethylene, flagellin, jasmonic acid, night, reactive oxygen species, salicylic acid

## Abstract

The first line of plant defence responses against pathogens can be induced by the bacterial flg22 and can be dependent on various external and internal factors. Here, we firstly studied the effects of daytime and ethylene (ET) using *Never ripe* (*Nr*) mutants in the local and systemic defence responses of intact tomato plants after flg22 treatments. Flg22 was applied in the afternoon and at night and rapid reactions were detected. The production of hydrogen peroxide and nitric oxide was induced by flg22 locally, while superoxide was induced systemically, in wild type plants in the light period, but all remained lower at night and in *Nr* leaves. Flg22 elevated, locally, the ET, jasmonic acid (JA) and salicylic acid (SA) levels in the light period; these levels did not change significantly at night. Expression of *Pathogenesis-related 1* (*PR1*), *Ethylene response factor 1* (*ERF1*) and *Defensin* (*DEF*) showed also daytime- and ET-dependent changes. Enhanced *ERF1* and *DEF* expression and stomatal closure were also observable in systemic leaves of wild type plants in the light. These data demonstrate that early biotic signalling in flg22-treated leaves and distal ones is an ET-dependent process and it is also determined by the time of day and inhibited in the early night phase.

## 1. Introduction

Plants have co-evolved with microbes, including bacterial and fungal pathogens, under diverse environmental stimuli, which have resulted in the formation of a number of signalling pathways regulating local and systemic defence responses in higher plants. Transmembrane pattern recognition receptors (PRR) of plants recognize attacks of a wide variety of these pathogens by detecting microbial- or pathogen-associated molecular patterns (MAMPs or PAMPs), promote well-characterised processes, such as changes in cytosolic free calcium level, production of reactive oxygen species (ROS) and activation of protein kinases, and cause rapid closure of the epidermis-localised stomata as a part of pattern-triggered immunity (PTI), as well as inducing the synthesis of antimicrobial compounds and changes in cell wall composition [1,2]. Defence responses of plants are regulated by several phytohormones which orchestrate the production of defensive proteins (e.g., pathogenesis-related (PR) proteins, defensins) or secondary metabolites (e.g., phytoalexins). The principal hormonal defence regulators are salicylic acid (SA), jasmonic acid (JA), ethylene (ET) and abscisic acid (ABA) [3,4]. SA plays a pivotal role in many plant–pathogen interactions, typically in defence against biotrophs and in the biotrophic stage of hemibiotrophs, by inducing localized death of infected cells, the hypersensitive response (HR) and the systemic acquired resistance (SAR) in long-distance tissues [5,6,7,8,9,10,11]. The other important defence regulators are JA and ET, which take part, generally, in defence responses against necrotrophic pathogens [12,13,14]. At the same time, ET functions as a hormonal fine regulator of defence responses in a time- and concentration-dependent manner [15,16], while ABA plays a crucial role in stomatal closure, serving as the first line of defence against pathogen entry [17]. Meanwhile, the hormonal crosstalk-mediated responses of plants to pathogens are highly dependent on internal and external factors, such as the circadian regulation, or environmental conditions, such as light, temperature or water availability [18,19,20,21,22]. Light is one of the major external factors that influences not only the optimal plant growth and development but also plant defence responses [21,23,24,25,26]. Thus, the presence or absence, period, quality, intensity and timing of light can alter and influence plant defence responses and metabolism [27]. Plants have evolved various types of photoreceptors, namely, the red/far-red light-sensing phytochromes (phyA–phyE), the blue light-sensing cryptochromes (cry1-3) and phototropins (phot1 and phot2), the Zeitlupe family members (ZTL, FKF1, LKP2) and the UV-B receptor UVR8, which are capable to perceive and mediate responses to the quantity and quality of light [28,29]. These photoreceptors are also important components in the resistance to various pathogens [21]. Both HR and SAR are light-dependent and mediated by phyA and phyB photoreceptors [18,20]. Moreover, it was found that the defence responses of plants are attenuated and delayed in the dark [24,30]. Similarly to plants, several physiological features of plant–pathogen bacteria, especially motility, adhesion and virulence, are also influenced by light. However, in contrast to plants, increased infection capability was detected under darkness in the case of *Pseudomonas syringae* pv. syringae B728a (*Psy*) [31,32], *Pseudomonas syringae* pv. tomato (*Pto*) [33,34], *Pseudomonas cichorii* JBC1 [35,36], *Agrobacterium fabrum* [37] and *Xanthomonas campestris* [38]. Thus, plants can be subjected to a greater challenge by many bacterial plant pathogens at night in the dark, than during the day [24]; however, the light-dependent defence responses of plants, especially in crops, are less investigated.

In addition, the basic levels of SA and JA, thus plant immune responses, are regulated by the circadian clock [22,39]. *TIME FOR COFFEE* (*TIC*), a night-expressed clock gene, inhibits JA signalling in the evening and contributes to a stronger JA responsiveness in the morning. The peak of JA accumulation is in the middle of the day, while the concentration of SA is the highest around the middle of the night, because the expression of the *ISOCHORISMATE SYNTHASE 1* gene, a key enzyme of SA biosynthesis, is driven by the evening-phased clock transcription factor *CIRCADIAN CLOCK ASSOCIATED 1* (*CCA1*) *HIKING EXPEDITION* (*CHE*) [40]. In addition, the early-night-expressed *TIMING OF CAB EXPRESSION 1* (*TOC1*) and the dawn-expressed *CCA1* also control the ET emission, which was peaked around midday of the light period in *Arabidopsis* seedlings [41]. However, ET signalling was not mediated by the phase or period of circadian rhythms during the growth and development of seedlings [41]. Based on the results of molecular studies, understanding the role of defence hormones in plant responses to pathogen infection in different day/night-time provides an important perspective for plant stress physiology and plant protection.

Infection of bacterial pathogens can be mimicked by elicitor molecules, such as the PAMP molecule flg22, which comprises 22 amino acids from a conserved region of the N-terminus of the bacterial flagellin [42]. Flg22 is perceived by the RLK receptor complex FLS2-BAK1 [43,44] which phosphorylates the kinase BIK1, which than activates the plasma membrane-localized NADPH oxidase AtRBOHD in *Arabidopsis* [45,46]. In tomato, the FLS2 receptor was also described to sense flg22 and other epitopes of bacterial flagellin derived from *Escherichia coli*, flg15E coli. Both epitopes resulted in significant ROS production and ET emission in tomato [47]. In addition, others characterised the FLS3 receptor to sense other epitopes of flagellin, such as flgII-28, which also induces ROS-mediated signalling in tomato [48]. Increased ROS production by NADPH oxidase promotes the activation of plasma membrane-localized Ca^2+^ channels in guard cells within minutes in vitro [49,50,51] and, subsequently, of the SLAC1 anion channel and aquaporin PIP2;1, which leads to rapid stomatal closure upon flg22 treatment [52,53,54]. Not only RBODH, but also mitogen-activated kinases MPK3 and MPK6 are activated upon flg22, thus regulating the stomatal closure [50,55,56]. Furthermore, flg22 induces callose deposition several hours later—already after 8–12 h—in the treated leaves as a part of defence responses [57,58,59].

The role of defence hormones, especially the role of ET in plant responses upon flg22, is contradictory. Analysis of mutants and transgenic plants related to SA, JA and ET sensing and signalling resulted that the tested genes are not required for flg22-induced bacterial disease resistance in *Arabidopsis* after 24 h [44]. At the same time, it was found that flg22 elevated the expression of the SA-mediated marker gene *PR1* after 24 h in *Arabidopsis* [60]. Later, it was measured that flg22 induced both SA and JA accumulation after 8 h [61]. In addition, flg22 activates the production of ET within 2 h and triggers a rapid oxidative burst in tomato leaves [42]. The study of Denoux et al. [62] showed, for the first time, that the treatment with flg22 triggers a fast (1 h) and transient response in the early stages of multiple defence signalling pathways mediated by all, SA, JA and ET. Early responses are associated with JA and late responses are mostly mediated by SA. In addition, the activation of ET biosynthesis was also detected in flg22-treated *Arabidopsis*. Application of flg22 induced the transcript accumulation of *Amino–cyclopropane–carboxylate synthase* (*ACS*) genes already after 1 h, which catalyses the rate-limiting step of ET biosynthesis [62]. Others also found that expression of ACS genes (*ACS2*, *ACS6* and *ACS8*) was elevated by flg22 infiltration into *Arabidopsis* leaves [63]. Moreover, significant ET emission induced by different concentration of flg22 was also described, after 2 h, in tobacco leaves [64]. The role of ET in defence responses upon flg22 was firstly described by Mersmann et al. [65]. Based on the investigation of ethylene-insensitive *etr1-1* and *ein2-1* receptor and signalling mutants, the expression of *FLS2* was under the control of basal ET, which influenced the *FLS2* steady-state levels. Moreover, several ET response elements can be found in the *FLS2* promoter region [65]. The main findings of this research are that flg22-triggered ROS production remained significantly lower, but activation of MPK3 and MPK6 remained unaltered in the ET-insensitive mutants. In addition, stomatal closure caused by flg22 was not detected in these ET-insensitive mutants. These data suggest that ET plays a significant role in ROS production and stomatal closure, locally, at the early stages of infection [65]. Later, the positive effects of ET on *FLS2* expression were also confirmed by using the ET precursor ACC as a pre-treatment [66]. Others found that the activation of MPK6 was similar after 15 min in the case of flg22 treatments, but it was activated later, only after 10 min in *etr1-1* and *ein2-1* ET-insensitive mutants, as compared to the wild type (WT), where it was already activated after 5 min [67]. Interestingly, activation of MPK6 caused the phosphorylation of ACS2 and ACS6, leading to the accumulation of the ACS protein, thus elevating ACS activity and ET production in *Arabidopsis* [68]. Although the role of ET in flg22-induced fast defence responses has been carefully described, the effects of gaseous ET in the response of distal leaves of intact plants were not investigated. Moreover, the local and systemic responses of plants could be dependent on the presence/absence of light and day/night-time. Thus, understanding the role of these factors in ET-mediated local and systemic defence responses could be significant, because the flg22-induced gene expression is highly dependent on the presence of light [69]. Not only ET production and signalling [70] but also nitric oxide (NO), as well as ROS production via NADPH oxidase and ROS metabolism, are different under darkness [71,72,73].

Using ET receptor and signalling mutants provides a more precise analysis of the physiological and molecular functions of ET. The ET receptor SlETR3 in tomato is known as *Never ripe* (*Nr*) [74], which is the ortholog of the *Arabidopsis* ETR1 receptor [75]. This mutant exhibits insensitivity to ET not only in fruit ripening, but in all vegetative tissues. However, *Nr* is able to produce ET upon pathogen attack, indicating that the mutants are not impaired in ET biosynthesis [76]. At the same time, the mutation in ET sensing results in complex physiological and biochemical changes in *Nr* vegetative tissues, compared to WT [77], as well as in significant changes in basic phytohormone levels, such as SA [78] or ABA [79], influencing the outcome of defence responses

In this work, our aim is to investigate the daytime- and ET-dependent effects of flg22 on intact leaves of tomato plants. The local and systemic daytime- and ET-dependent ROS/NO production and stomatal closure, as well as defence-related phytohormone levels (ET, JA and SA) and signalling, based on the expression profile of their response marker genes, were also analysed in leaves of intact WT and ET receptor mutant *Never ripe* (*Nr*) plants.

## 2. Results

### 2.1. Flg22 Elevates ROS Production in a Daytime- and ET-Dependent Manner

**Figure 1:**
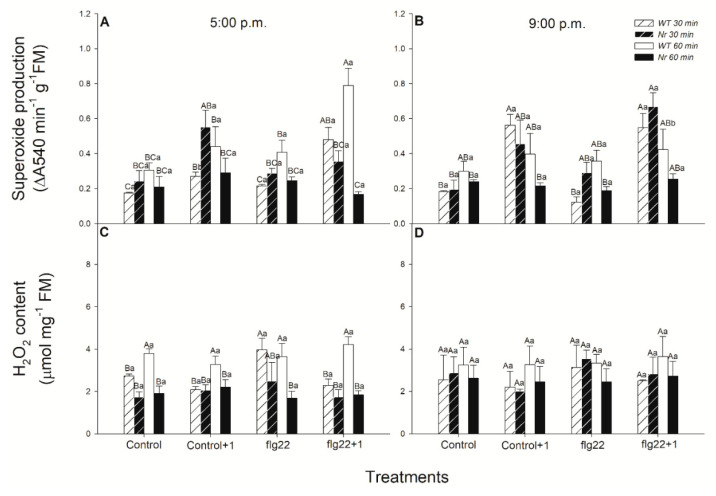
Changes in the superoxide production (**A**,**B**) and H_2_O_2_ content (**C**,**D**) in leaves of intact wild type (WT; white columns) and ethylene-insensitive *Never ripe* (*Nr*; black columns) tomato plants treated foliar using a squirrel-hair brush with 5 μg mL^−1^ of flagellin (flg22) in the late afternoon under light (at 5:00 p.m.) or at night under darkness (at 9:00 p.m.). Measurements were carried out 30 min and 60 min after treatments. Whole leaves from different leaf levels and genotypes were ground and superoxide production, as well as H_2_O_2_ content, were determined using spectrophotometric methods. Means ± SE, *n* = 3. Means were analysed by two-way ANOVA; significant differences among the data were analysed by Duncan’s test. Mean values significantly different at *p* < 0.05 are indicated by different letters, upper case letters indicate the effects of the treatment at the same time of day and lower case letters indicate the effects of daytime under the same treatment. (Control, treatment with sterile distilled water; Control+1, untreated distal leaf level to the control; flg22, treatment with 5 μg mL^−1^ of flagellin dissolved in sterile distilled water; flg22+1, untreated distal leaf level to the flg22-treated one).

To test the ET-dependent effects of 5 μg mL^−1^ of flg22 on defence responses, changes in ROS production (superoxide and hydrogen peroxide) in leaves of intact WT and ethylene-insensitive *Never ripe* (*Nr*) plants were firstly investigated using spectrophotometric methods. Measurements were carried out 30 and 60 min after the flg22 treatments (at 5:00 p.m. in the light period and at 9:00 p.m. in the dark period) to reveal the role of daytime-dependent effects of the bacterial elicitor in treated (6th leaf levels from the shoot apex, local effect) and in distal, systemic leaves (5th leaf levels from the shoot apex) of intact tomato plants. The treatment with flg22 only slightly elevated the superoxide production locally in WT plants at the selected two time points, but a significant increase was detected in distal leaves to flg22-treated ones, one hour later, measured in the light period (Figure 1A). At the same time, this increment in superoxide production was neither detected in the evening (Figure 1B) nor in *Nr* leaves at all (Figure 1A,B), suggesting a daytime- and ethylene-dependent regulation of superoxide production. Other ROS, such as H_2_O_2_ levels, were basically lower in *Nr* leaves (Figure 1C,D). Treatment with flg22 significantly increased this after 30 min in WT leaves, but it did not change upon flg22 one hour later in WT plants (Figure 1C). H_2_O_2_ levels did not change in leaves of *Nr* plants nor in the evening after the elicitor treatments (Figure 1C,D).

### 2.2. NO Accumulation Induced by flg22 Is Mediated by ET and Light

**Figure 2:**
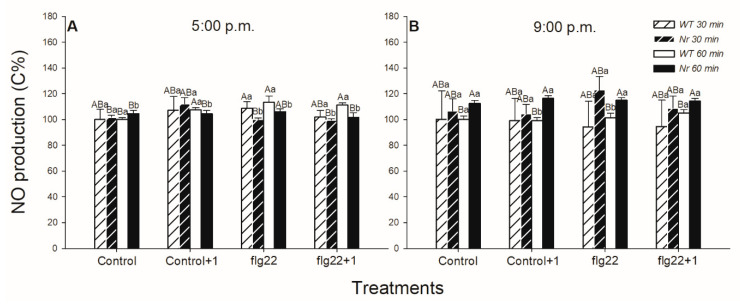
Changes in the nitric oxide (NO) production in leaves of intact wild type (WT; white columns) and ethylene-insensitive *Never ripe* (*Nr*; black columns) tomato plants treated foliar using a squirrel-hair brush with 5 μg mL^−1^ of flagellin (flg22) in the late afternoon under light (at 5:00 p.m.) (**A**) or at night under darkness (at 9:00 p.m.) (**B**). Measurements were carried out 30 and 60 min after treatments. Leaf discs prepared immediately from the whole leaves from the different leaf levels and genotypes were incubated in DAF-FM DA fluorescent dye; then, fluorescence intensities were determined by microscope. Means ± SE, *n* = 3. Means were analysed by two-way ANOVA; significant differences among the data were analysed by Duncan’s test. Mean values significantly different at *p* < 0.05 are indicated by different letters, upper case letters indicate the effects of the treatment at the same time of day and lower case letters indicate the effects of daytime under the same treatment. (Control, treatment with sterile distilled water; Control+1, untreated distal leaf level to the control; flg22, treatment with 5 μg mL^−1^ of flagellin dissolved in sterile distilled water; flg22+1, untreated distal leaf level to the flg22-treated one).

The ET- and daytime-dependent production of NO, as another cell signalling component and effector molecule in defence responses, was also analysed in the leaves of WT and *Nr* plants using the fluorescent staining method. NO production did not change significantly after the 30-minute-long flg22 exposure, but it increased after 60 min locally and in the distal leaves of WT plants upon flg22; however, it was basically higher in the upper-leaf level from the control (Figure 2A). Nevertheless, levels of NO did not change significantly in *Nr* leaves, where it was basically higher (Figure 2). At the same time, there was no significant detectable change in the generation of NO in WT leaves at night, in the dark (Figure 2B).

### 2.3. Flg22 Contributes to Local Accumulation of ET, JA and SA, Which Is Inhibited at Night

**Figure 3:**
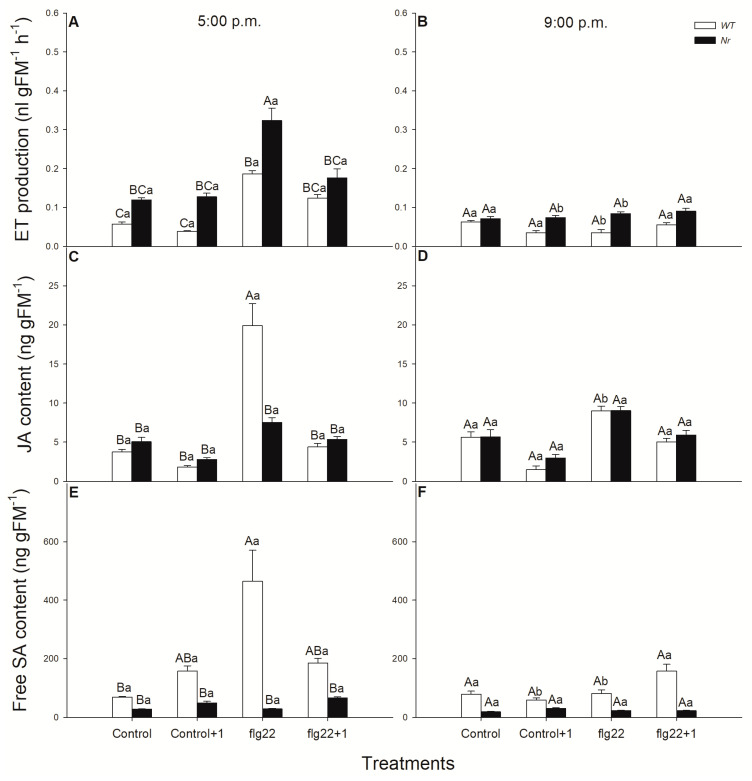
Changes in the ethylene (ET) emission (**A**,**B**), jasmonic acid (JA) content (**C**,**D**) and salicylic acid (SA) content (**E**,**F**) in leaves of intact wild type (WT; white columns) and ethylene-insensitive *Never ripe* (*Nr*; black columns) tomato plants treated foliar using a squirrel-hair brush with 5 μg mL^−1^ of flagellin (flg22) in the late afternoon under light (at 5:00 p.m.) or at night under darkness (at 9:00 p.m.). Measurements were carried out one hour after treatments (at 6:00 p.m. and 10:00 p.m.). Whole leaves from the different leaf levels and genotypes were collected and immediately frozen in liquid nitrogen; then, hormone levels were determined by chromatography methods. Means ± SE, *n* = 3. Means were analysed by two-way ANOVA; significant differences among the data were analysed by Duncan’s test. Mean values significantly different at *p* < 0.05 are indicated by different letters, upper case letters indicate the effects of the treatment at the same time of day and lower case letters indicate the effects of daytime under the same treatment. (Control, treatment with sterile distilled water; Control+1, untreated distal leaf level to the control; flg22, treatment with 5μg mL^−1^ of flagellin dissolved in sterile distilled water; flg22+1, untreated distal leaf level to the flg22-treated one).

The activation of defence responses is highly regulated by various phytohormones. In the next experiments, the levels of ET, JA and SA were measured using chromatography methods after flg22 treatments in WT and *Nr* leaves. Flg22 treatment in intact leaves of tomato significantly promoted ET emission locally both in WT and *Nr* plants in the late afternoon (Figure 3A). Surprisingly, the production of this gaseous phytohormone did not change in the early night period under darkness (Figure 3B). Similar changes in JA contents were observed in WT leaves treated with flg22 at 5:00 p.m. (Figure 3C), but, in *Nr* leaves and in the evening, JA content was not elevated by the bacterial elicitor treatments (Figure 3D). SA levels increased, locally, after flg22 treatments at the end of the light period and it was basically higher in the upper leaf level from the control (Figure 3E). At the same time, the accumulation of SA was not detected at night in any of the genotypes (Figure 3F). Moreover, SA contents were basically lower in *Nr* leaves, as compared to WT ones; furthermore, flg22 treatments did not cause any significant changes in SA levels in the mutant leaves (Figure 3E,F).

### 2.4. Expression of ERF1, DEF and PR1 upon flg22 Is Determined by Daytime and ET Both Locally and Systemically

**Figure 4:**
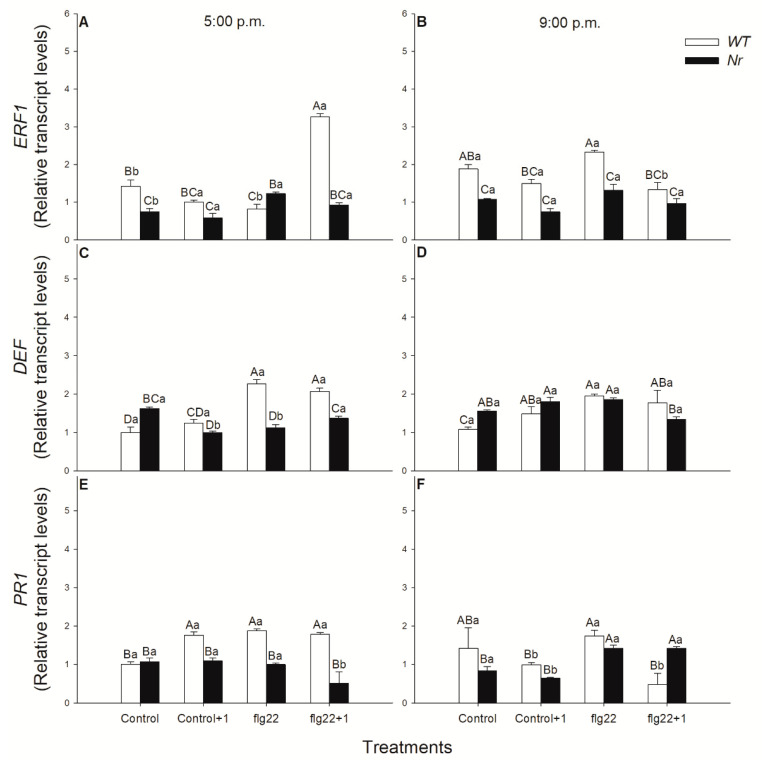
Changes in the relative transcript levels of *ERF1* (**A**,**B**), *DEF* (**C**,**D**) and *PR1* (**E**,**F**) in leaves of intact wild type (WT; white columns) and ethylene-insensitive *Never ripe* (*Nr*; black columns) tomato plants treated foliar using a squirrel-hair brush with 5 μg mL^−1^ of flagellin (flg22) in the late afternoon under light (at 5:00 p.m.) or at night under darkness (at 9:00 p.m.). Measurements were carried out one hour after treatments (at 6:00 p.m. and 10:00 p.m.). Whole leaves from the different leaf levels and genotypes were collected and immediately frozen in liquid nitrogen; then, after RNA extraction, gene expression was analysed using qRT-PCR. Means ± SE, *n* = 3. Means were analysed by two-way ANOVA; significant differences among the data were analysed by Duncan’s test. Mean values significantly different at *p* < 0.05 are indicated by different letters, upper case letters indicate the effects of the treatment at the same time of day and lower case letters indicate the effects of daytime under the same treatment. (Control, treatment with sterile distilled water; Control+1, untreated distal leaf level to the control; flg22, treatment with 5μg mL^−1^ of flagellin dissolved in sterile distilled water; flg22+1, untreated distal leaf level to the flg22-treated one).

Phytohormone-mediated signalling can be analysed using marker genes, such as *ERF1*, *DEF* and *PR1*. Relative transcript levels of these genes were detected after RNA extraction from the flg22-treated and systemic leaves via quantitative real-time PCR analysis. Treatment with flg22 induced the ET response factor *ERF1* expression within 1 h in the distal leaves of the elicitor-treated ones of WT plants in the late light phase (Figure 4A). At the same time, these changes in *ERF1* transcript levels were not observable in the dark at 9:00 p.m., neither in WT nor in *Nr* leaves (Figure 4B). Moreover, exposure to flg22 elevated the transcript levels of *DEF* both locally and systemically in WT leaves and was independent from the daytime of the treatments, but the effect of the elicitor seemed to be more significant in the light (Figure 4C,D). Similar to *DEF*, the expression of the SA-related *PR1* was also significantly promoted locally by flg22 in WT leaves, as compared to the control, but only in the light period (Figure 4E).

### 2.5. Flg22-Induced Local and Systemic Stomatal Closure Is Inhibited in Nr Mutants and at Night

**Figure 5:**
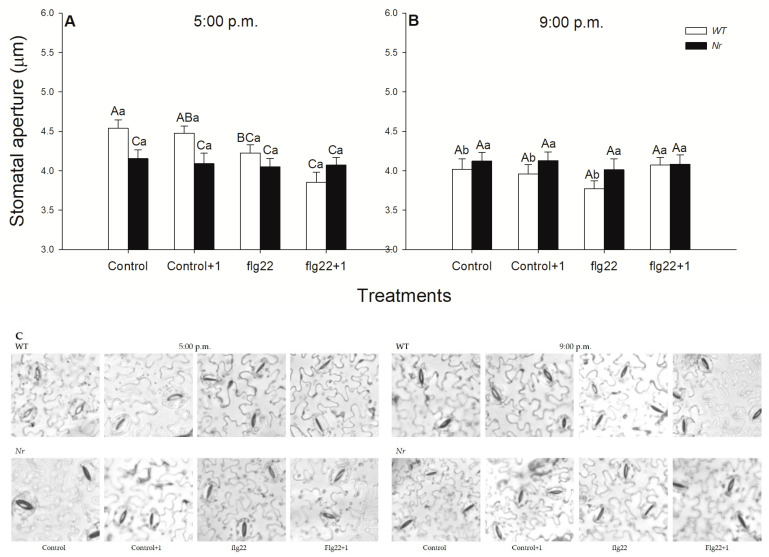
Changes in the size of stomatal apertures on the abaxial epidermal strips of intact wild type (WT; white columns) and ethylene-insensitive *Never ripe* (*Nr*; black columns) tomato plants treated foliar using a squirrel-hair brush with 5 μg mL^−1^ of flagellin (flg22) in the late afternoon under light (at 5:00 p.m.) (**A**) or at night under darkness (at 9:00 p.m.; (**B**). Measurements were carried out one hour after treatments (at 6:00 p.m. and 10:00 p.m.). Epidermal peels were prepared immediately from the whole leaves from the different leaf levels and genotypes; then, microscopic photos were taken rapidly and the stomatal pore size was determined digitally (**C**). Means ± SE, *n* = 3. Means were analysed by two-way ANOVA; significant differences among the data were analysed by Duncan’s test. Mean values significantly different at *p* < 0.05 are indicated by different letters, upper case letters indicate the effects of the treatment at the same time of day and lower case letters indicate the effects of daytime under the same treatment. (Control, treatment with sterile distilled water; Control+1, untreated distal leaf level to the control; flg22, treatment with 5 μg mL^−1^ of flagellin dissolved in sterile distilled water; flg22+1, untreated distal leaf level to the flg22-treated one).

Finally, the ET- and daytime-dependent effects of flg22 on plant physiological responses were investigated based on the responses of stomata using microscopic detection. Interestingly, while flg22 treatment significantly decreased, locally, the stomatal pore size at 5:00 p.m. in the light, it did not induce significant stomatal closure when compared to the control at 9:00 p.m. in the dark (Figure 5). In addition, flg22 treatments caused significant stomatal closure in distal leaves to flg22-treated ones 1 h after the elicitor treatment in the afternoon (Figure 5A), but not at night (Figure 5B). In contrast to these, the size of the stomatal aperture in ethylene-insensitive *Nr* plants did not change significantly upon flg22 in any of the times of day (Figure 5).

## 3. Discussion

Successful defence mechanisms of plants are related to the timing of molecular and physiological responses regulated by several phytohormones, such as ET [3,15,16]. However, these processes are dependent on external factors, such as the presence/absence of light, or on internal factors, such as the effects of the circadian clock [22,27,80]. At the same time, it was found that the ET-mediated signalling is not dependent on circadian rhythms during plant growth and development [41]. In contrast, ET was described as a hormonal fine regulator beside JA and SA, but these hormone levels are highly regulated by the circadian clock [16,22]. Nevertheless, the action of ET was highly dependent on daytime and on its concentration, as well as on light intensity [81,82,83]. In this article, the ET- and daytime-dependent effects of the bacterial elicitor flg22 were examined locally and systemically using intact tomato plants, which can provide new results to understand the complexity of early defence responses of plants. Although the concentration-dependent effects of bacterial flagellin cannot be disclosed in the activation of defence responses of plants, a widely used micro-molar concentration of flg22 was selected for these experiments [49,53,56]. While it is hard to distinguish the direct effect of external light/darkness from the internal effect of circadian rhythm on plants, the two closest time points were chosen in the late light and early dark period of the day (p.m. 5:00 and 9:00 p.m.) for the experiments. In the afternoon, stomata start to close and finish to accumulate photoassimilates [84]. At the same time, circadian-regulated JA levels show a peak [85]; thus, signalling of stomatal closure and defence could be different, compared with those measured in the morning. Nevertheless, to mimic the natural environmental conditions, we wanted to choose a time-point in the early night period to compare and describe plant defence responses, instead of using artificial darkening, because most of the plant bacteria are more active at night [33]. Time-point for nocturnal investigation was selected at 9:00 p.m., 3 h after the end of the light period, when light-dependent processes of photosynthesis and active phytochrome signalling are inactive [86,87]. At the same time, in the case of these selected time points, there could be no significant differences in the availability of carbohydrate and starch providing metabolic energy [88], which could influence the defence responses of plants. The flg22-induced local and systemic defence responses were investigated after 30 and 60 min of the bacterial elicitor treatment. Based on previous results, these time-periods seem to be optimal for the activation and detection of the rapid molecular and physiological changes in intact plants, such as ROS production [53,89,90], activation of defence- and phytohormone-related genes [62,69] and stomatal closure [52]. These selected time-points after the bacterial elicitor treatments provide the possibility for the comparison of the daytime-dependent local and systemic effects of flg22-induced defence responses detected in intact tomato plants.

It is well-known that, after the recognition of pathogens by detecting MAMPs or PAMPs by PRRs, rapid production of ROS is induced [1]. The plasma membrane-localized flavoenzyme NADPH oxidase, also known as respiratory burst oxidase homologue (RBOH), can translocate electrons from cytosolic NADPH to oxygen, leading to the generation of superoxide anion radicals. This rapid production of superoxide plays a crucial role in the local activation of intracellular signalling and in facilitating rapid signal propagation, which is prerequisite for mounting systemic defences at distal sites of the whole plant [91,92,93]. Based on our results, the superoxide production was promoted but it was not significant after the flg22 treatment in WT plants in the light period; however, at the same time, a significant increase was detected in superoxide production in distal leaves. This superoxide production and H_2_O_2_ levels were lower in *Nr* leaves, as compared to WT ones. In addition, superoxide production did not change in the systemic leaves of *Nr*, as compared to WT, confirming the role of ET in the systemic superoxide production after flg22 exposure. Under darkness, in the evening, significant changes were not detectable neither in flg22-treated WT nor in *Nr* leaves. Thus, these results confirmed the daytime- and ET-dependent superoxide production after the bacterial elicitor flg22 exposure in intact tomato plants. Earlier, it was described, using leaf disks (with a luminol-based assay in a microplate), that flg22 induced a rapid ROS burst within minutes [47,89,90,94,95]. This in situ detection of ROS suggests that the peak in superoxide production was a fast response of plant leaves locally upon flg22 treatment. Based on our investigation, increased levels of superoxide were detected only in the systemic leaves of intact WT plants and were missing at night and in *Nr* leaves. Activation of NADPH oxidase and fast superoxide production result in an “ROS wave” and rapid systemic response in distal parts of plants from the stress stimuli [96]. It is known that ET, in a concentration-dependent manner, can induce a rapid production of superoxide and H_2_O_2_ in the leaves [82]. Mersmann et al. [65] found that ROS production after flg22 exposure was diminished in ET-insensitive receptor mutant seedlings and confirmed that ET plays a crucial role in the early stage of the plant immune response. Moreover, a dark exposure can also delay and inhibit the activity of NADPH oxidase, which contributes to the first, priming oxidative burst after pathogen infection [71]. Thus, in the dark, at night, the inhibition and/or decreased activity of NADPH oxidase and the “ROS wave” could contribute to the delay of defence responses both locally and systemically. In addition, it is known that a number of (30%) genes induced by flg22 require light for their rapid expression based on whole-transcriptome analysis of 30-minute-long flg22-treated *Arabidopsis* seedlings [69]. Thus, the absence of light could also contribute to delayed or inhibited defence responses of plants on a molecular level by influencing sensing and signalling of the stress stimuli by regulating ROS- and phytohormone metabolism. It can be concluded that ET plays a role in the systemic response of tomato plants by superoxide production in the light period.

Dismutation of the superoxide radical into molecular oxygen and hydrogen peroxide is mediated by superoxide dismutase, an enzyme also regulated by phytohormones such as ET or SA [72,78]. H_2_O_2_ content increased significantly 30 min after the flg22 treatment locally in WT leaves, but it did not change in the evening hours and in *Nr* plants. H_2_O_2_ production is also part of the local response of leaves following flg22 exposure, which plays a crucial role in a fast defence response by regulating the redox state of cells and expression of many defence-related genes [97]. Deger et al. [52] also observed a peak in H_2_O_2_ production after minutes in mesophyll tissue of *Arabidopsis* measured with a platinum microdisk electrode. It is well known that H_2_O_2_ can act together with SA and ET in a self-amplifying feedback loop, where SA and ET induce H_2_O_2_ production and H_2_O_2_ enhances the accumulation of both hormones [98], but it has to be mentioned that the dark can also decrease H_2_O_2_ accumulation [71]. Thus, both ET and the dark can modulate ROS levels locally and systemically upon flg22 treatment and influence the activation of defence responses of plants. It can be concluded that H_2_O_2_ production 30 min after flg22 treatment in the light period can play a significant role in the activation of local phytohormone-mediated defence responses of intact tomato plants. In addition, superoxide production, as a result of NADPH oxidase activity [99], plays a crucial role in cell–cell interaction and establishment of the “ROS wave”, thus the activation of a rapid systemic response in the distal part of intact plants [96]. At the same time, the role of antioxidant machinery and the effects of phytohormones on it cannot be disclosed in the regulation of superoxide/H_2_O_2_ levels at these time points [78,100].

ROS can interplay with NO in a variety of ways and it is a crucial partner in determining the cell fate or in the signalling responses in a number of physiological and stress-related conditions [64,101]. Based on our results, NO production increased locally only 1 h after flg22 treatment in WT plants in the light, but not at night and in *Nr* leaves. These results confirmed that the active ET signalling and the presence of light can determine the local NO production in the intact leaves of tomato plants influencing defence responses. Earlier, it was observed that flg22 triggered NO production in leaf discs of different maize lines after 2 h [102], but its effect in the development of systemic responses was not investigated. However, NO did not take part in the rapid systemic response of tomato after 1 h of flg22 treatment, based on our results. It is also known that the activity of nitrate reductase, which is associated with the direct synthesis of NO, is inhibited by the dark [73]. Based on our results, NO levels did not change after flg22 treatments in WT at night either. Thus, NO could be an integral part of local defence responses of plants in the light, whose absence could contribute to delayed or inhibited defence responses under darkness at night. It is also well known that both ROS and NO take part in the induction of rapid stomatal closure, thus mediating fast defence responses of plants [49,64]. These reactive species could also contribute to the stomatal closure upon flg22 in tomato in the light phase. Moreover, it is well known that NO plays a role in post-translational modification of some defence-related proteins and it is in tight interaction with ET, JA and SA biosynthesis and signalling during the daytime [103,104,105,106].

ET is a fine regulator of JA/SA-mediated short- and long-term defence responses of plants. However, the rapid local and systemic effects of ET are less-known, especially its daytime-dependent role, while the function of other mobile signals of SAR in phloem is well-characterised [105]. It was earlier found that treatment with flg22 already initiated the expression of ET biosynthetic *ACS* genes within 1 h in *Arabidopsis* and, in parallel, early stages of multiple defence signalling pathways mediated by all, SA, JA and ET, were activated in the daytime [62]. Others also measured significant ET emission after flg22 exposure within hours in the light [42,64], but the effects of ET in the response of distal leaves of intact plants were not investigated. Here, we also observed that flg22 significantly promoted ET production locally both in WT and *Nr* plants in the late afternoon, but ET emission did not change at night under darkness. This flg22-induced ET production can contribute to elevated *ERF1* expression in the distal leaves of WT plants within 1 h, which was not observed at night and in *Nr* leaves. This can be dependent on the ROS and NO in these leaves, based on our results, because both can enhance the production of ET [98,103]. This observation provides evidence, for the first time, that ET can not only play a role locally, but also systemically, in the fast and rapid response of defence in the light period. One-hour-long flg22 treatment significantly elevated *ERF1* expression, which can promote the expression of the ET -and JA-regulated defence response defensin gene, *PDF1.2*, in *Arabidopsis* [62]. Moreover, it was also found that ET and JA play a role in the rhizobacteria-induced systemic resistance (ISR), which enhanced the defensive capacity of plants against the bacterial pathogen *Pseudomonas syringae* by inducing ET production within hours [107]. The role of ET in the systemic response of tomato against another bacterial pathogen, *Xanthomonas campestris*, was also described beside JA, where a rapid ET- and pathogenesis-related gene induction was measured [108]. Moreover, local and systemic induction of *ERF*s were observed after *Pseudomonas syringae* or *Xanthomonas translucens* infection in barley [109]. Here, we found that flg22 induced, locally, both JA and *DEF* transcript accumulation only in WT, not in *Nr* plants. These results suggest that ET plays a role in the JA-mediated signalling under flg22 exposure locally. Surprisingly, in contrast to *Arabidopsis*, systemic immunity was not associated with the expression of *PR1* or the local or systemic accumulation of SA in this plant species [109]. In addition, elevated levels of both SA and *PR1* transcripts were measured locally after flg22 treatment and were not detected at 21:00 and in *Nr* leaves. These suggest the significant regulatory role of ET in local flg22-induced SA signalling in the light period. Earlier, it was found that flg22 increased the expression of SA-mediated marker gene *PR1* [60] and it induced both SA and JA accumulation in *Arabidopsis* [61]. It can be concluded that flg22 triggers the accumulation of all defence hormones and their signalling locally within one hour in intact plants, but systemic responses after flg22 treatments were recorded based on the analysis of the key response genes of the investigated defence hormones in the daytime. Namely, higher expression of *ERF1* and *DEF* was measured in systemic leaves of WT plants in the light period, the transcript levels of which did not change in *Nr* leaves, confirming the role of ET in rapid systemic response upon flg22 in this time of day. These results verified the possible daytime-dependent priming role of ET in distal leaves to flg22-treated ones, similarly to ISR [109]. In addition, the fine regulator role of ET in the defence-related phytohormone network was also confirmed by our result in the locally flg22-treated leaves in the light.

Closure of stomata in response to pathogens is one of the earliest events of plant defence responses [5,110]. Stomatal movements are not only regulated by phytohormones (e.g., ET, JA and SA), ROS and NO, but also by the environment, such as light or dark [53,110,111]. Earlier, it was observed that flg22 inhibited the light-induced stomatal opening in epidermal peels of *Arabidopsis* [49], or it closed stomata of intact leaves of *Arabidopsis* within 50 min using the nanoinfusion technique [52]. While the ABA-independent stomatal closure was described [56], in which positive effects of ET [65], as well as the role of the circadian clock, were verified [112], the systemic effect of ET was not analysed. We also found significant stomatal closure after flg22 exposure in the treated and distal leaves of WT plants in the light phase, but neither in *Nr* leaves nor in the dark. Thus, ET not only promotes stomatal closure locally after flg22 treatment, but also systemically in the light period—but not at night. Based on the results, flg22-induced ET-dependent ROS and NO production could contribute to this rapid systemic stomatal closure. Taken together, these data highlight the importance of ET in the fast control of different immune responses locally and systemically at distinct times of the day in intact tomato plants.

## 4. Materials and Methods

### 4.1. Plant Material and Experimental Growth Conditions

Healthy seeds of wild type (WT) and ethylene-insensitive *Never ripe* (*Nr*) mutant tomatoes (*Solanum lycopersicum* L. cv. Ailsa Craig) were germinated in the dark (3 days at 26 °C) and seedlings were transferred to perlite for another two weeks. Healthy plants were placed in modified Hoagland nutrient solution as described earlier by Poór et al. [71] and they were grown for at least 5 weeks in a controlled environment (200 μmol m^−2^ s^−1^ photosynthetic photon flux density (PPFD; white LED (5700 K) illumination with supplementary FAR LEDs; PSI, Drásov, Czech Republic), 12/12 h light/dark period (light from 06:00 to 18:00 h and 12 h dark period during the remaining daytime), 24/22 °C day/night temperatures and relative humidity of 55–60%). The nutrient solution was changed three times a week. For the experiments, from 7- to 8-week-old intact plants in 8–9 developed leaf-level stages were used. 

### 4.2. Flagellin Treatments

**Figure 6:**
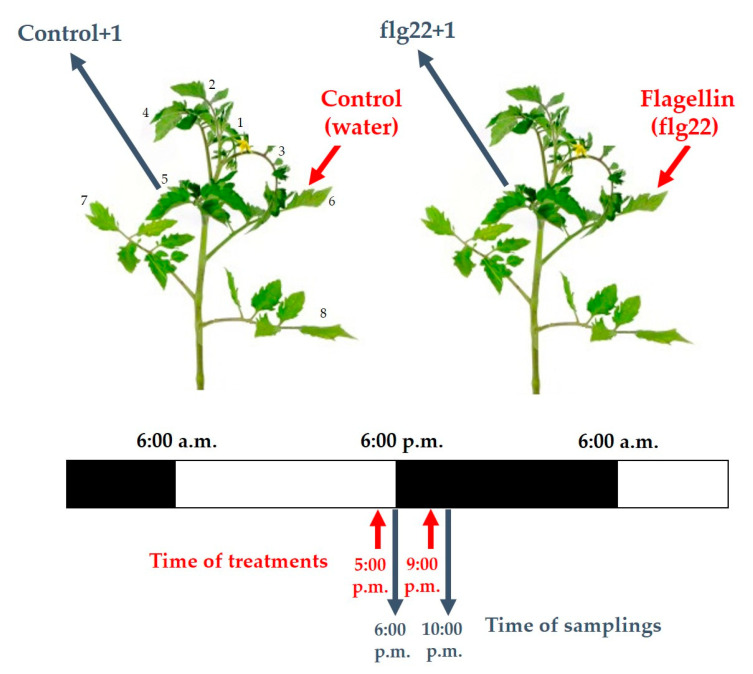
Experimental setup of flg22 treatments and time of samplings in intact tomato plants.

To study the daytime- and ethylene-dependent effects of flagellin in WT and *Nr* tomato plants, the abaxial side of leaves on the 6th leaf level of intact plants were treated with 5 μM flg22 (Genscript Biotech Corporation, Piscataway, NJ, USA), using a squirrel-hair brush, in the late afternoon (5:00 p.m.) and in the evening (9:00 p.m.), without wounding or disturbing the leaves [49,113,114]. Local and systemic defence responses of plants were detected after 30 and 60 min on the 6th leaf level from the shoot apex and on the distal 5th leaf level above the flg22-treated ones. As a control, sterile distilled water was used without flg22 (Figure 6).

### 4.3. Determination of Superoxide Production

A total of 100 mg of leaf tissues from the control and flg22-treated leaves of intact plants, as well as from the leaves above them, was homogenized in 1 mL of ice-cold sodium phosphate buffer (100 mM, pH 7.2) containing 1 mM sodium diethyldithiocarbamate trihydrate (SDDT; Sigma-Aldrich, St. Louis, MO, USA); then, the homogenate was centrifuged (13,000× *g* for 15 min at 4 °C). A volume of 300 µL of the supernatant was added to the reaction mixture which contained 650 µL of 100 mM sodium phosphate buffer (pH 7.2) and 50 µL of 12 mM nitroblue tetrazolium (NBT; Sigma-Aldrich, St. Louis, MO, USA). The absorbance of samples was determined before the incubation (A0) and after the 5-minute-long incubation period (AS) at 540 nm using a spectrophotometer (KONTRON, Milano, Italy). Superoxide production was calculated using the formula ΔA540 = AS − A0 and it was expressed as ΔA_540_ (min^−1^ g^−1^ fresh mass) [115]. Leaves of three different plants were used for sampling in the case of each treatment. Measurements were repeated 3 times using new plant generations.

### 4.4. Determination of Hydrogen Peroxide (H_2_O_2_) Content

Samples of 200 mg of leaf tissue as described at the determination of superoxide production were homogenized with 1 mL of ice-cold, 0.1% trichloroacetic acid (TCA), then centrifuged (11,500× *g* at 4 °C) for 10 min. A volume of 250 µL of the supernatant was added to the reaction mixture which contained 250 µL of 50 mM potassium phosphate buffer (pH 7.0) and 500 µL of 1 M potassium iodide (KI). After 10 min, the absorbance of the mixture was recorded at 390 nm with a spectrophotometer [116]. The amount of H_2_O_2_ was calculated using a standard curve of H_2_O_2_ solution (Sigma-Aldrich, St. Louis, MO, USA). Leaves of three different plants in three technical replications were used for sampling in the case of each treatment. Measurements were repeated 3 times using new plant generations.

### 4.5. Detection of Nitric Oxide (NO) Production

Leaf disks from the control and flg22-treated leaves of intact plants, as well as from the leaves above them, in three replications, were infiltrated with 10 μM 4-amino-5-methylamino-2′,7′-difluorofluorescein diacetate (DAF-FM DA) (Sigma-Aldrich, St. Louis, MO, USA) and dissolved in 10 mM Tris-HCl buffer (pH 7.4) under vacuum for 30 min in the dark at room temperature. After incubation, samples were rinsed twice with 10 mM Tris-HCl buffer solution (pH 7.4). The fluorescence intensity of DAF-FM DA was detected in the samples with a Zeiss Axiowert 200M-type fluorescence microscope (Carl Zeiss Inc., Jena, Germany) equipped with a high-resolution digital camera (Axiocam HR, Carl Zeiss Inc., Jena, Germany). Data were evaluated by the AXIOVISION REL. 4.8 software (Carl Zeiss Inc., Munich, Germany) [117,118]. Leaves of three different plants were used for sampling in the case of each treatment. Measurements were repeated 3 times using new plant generations.

### 4.6. Determination of Ethylene (ET) Emission

A total of 500 mg from the control and flg22-treated leaves of intact plants, as well as from the leaves above them, in six replications, was incubated in 25 mL gas-tight vials sealed with rubber serum caps at 25 °C for 1 h under dark condition. After incubation, 2.5 mL of the gas was removed manually from the tubes with a gas-tight syringe and injected into the gas chromatograph equipped with a flame ionization detector and a column packed with activated alumina (Hewlett-Packard 5890 Series II; Palo Alto, CA, USA). A set of ET standards was used to calculate the amount of ET emitted by the leaves [119]. Leaves of six different plants were used for sampling in the case of each treatment. Measurements were repeated 3 times using new plant generations.

### 4.7. Determination of Salicylic Acid (SA) and Jasmonic Acid (JA) Contents

One gram of the control and flg22-treated leaves of intact plants, as well as from the leaves above them, in five replications, was ground in liquid nitrogen and the powder was transferred to a centrifuge tube containing 2 mL of 70% methanol with 250 ng of O-anisic acid (oANI) and 25 mg of para-hydroxybenzoic acid (pHBA) for SA determination. The extract was centrifuged (10.000× *g* for 20 min) and the pellet was resuspended in 2 mL of 90% methanol; then, it was evaporated at room temperature under vacuum. A volume of 1 mL of 5% (*w*/*v*) TCA was added to the residual aqueous phase and the mixture was again centrifuged (15.000× *g* for 10 min). The supernatant was partitioned twice against 3 mL of 1:1 (*v*/*v*) mixture of ethyl acetate/cyclohexane and free phenolic acids accumulated in the upper organic layers. The aqueous phases with the methanol-soluble phenolics were acid hydrolysed. A total of 250 ng oANI, 25 μg pHBA and 1.3 mL 8 N HCl was added to the aqueous phase and incubated (60 min at 80 °C) before partitioning twice as above. The organic phases were evaporated under vacuum and resuspended in 1 mL of the HPLC initial mobile phase. Free SA content was determined using high-performance liquid chromatography (HPLC) on a reverse-phase column (Supelcosil ABZ Plus, 5 μm; 150 × 4.6 mm) at 25 °C (WATERS, Milford, MA, USA) and monitored with a UV/VIS detector (W474 scanning fluorescence detector, Waters, MA, USA) with excitation at 305 nm and emission at 407 nm [120].

JA content was determined according to Pál et al. [121], using ultra performance liquid chromatography with a Waters Acquity I class UPLC system (Milford, MA, USA). After that, the separation mass spectrometry detection was performed by a Waters Xevo TQXS (Milford, MA, USA) equipped with a Unispray Source (Milford, MA, USA). The Waters MassLynx 4.2 and TargetLynx software was used for data processing. Leaves of six different plants were used for sampling in the case of each treatment. Measurements were repeated two times using new plant generations.

### 4.8. Gene Expression Analyses by Quantitative Real-Time PCR

Total RNA was isolated from the control and flg22-treated leaves of intact plants, as well as from the leaves above them, using the TRI reagent method [122]. Leaves of three different plants were mixed and used for sampling in the case of each treatment. Measurements were repeated 3 times using new plant generations. DNase I (Fermentas UAB, Vilnius, Lithuania) was used for genomic DNA digestions and the first-strand cDNA was synthesized using MMLV reverse transcriptase (Fermentas UAB, Vilnius, Lithuania). Analysis of the expression pattern of the selected tomato genes was performed using quantitative real-time PCR (qRT-PCR; qTOWER Real-Time qPCR System, Analytik Jena, Jena, Germany). Tomato genes (*SlPR1* (Solyc01g106620): F, 5′-CATCCCGAGCACAAAACTATG-3′; R, 5′-CCCCAGCACCAGAATGAAT-3′. *SlERF1* (Solyc05g051200): F, 5′-GGAACATTTGATACTGCTGAAGA-3′; R, 5′-AGAGACCAAGGACCCCTCAT-3′. *SlDEF1* (Solyc07g007760): R, 5′-GGCACAATCCATTCGTTTCT-3′; F, 5′-TTGGTCCCATTTCAGTAGCC-3′) were mined from the Sol Genomics Network (SGN; http://solgenomics.net; last accessed on 31 January 2021) database [123]. The PCR reaction mixture contained 10 ng of cDNA template, 400–400 nM forward and reverse primers, 5 µL of Maxima SYBR Green qPCR Master Mix (2X) (Thermo Scientific, Waltham, MA, USA) and nuclease-free water in 10 μL volume (denaturation at 95 °C for 7 min, followed by 40 cycles of denaturation at 95 °C for 15 s and annealing extension at 60 °C for 60 s). To analyse the data, the qTOWER Software 2.2 (Analytik Jena, Jena, Germany) was applied [78]. The specificity of the qRT-PCR reaction was confirmed using melting curve analyses in all cases. Reference genes were the tomato 18S rRNA and elongation factor-1α subunit and the expression data were calculated by the 2^(−∆∆Ct)^ formula [124].

### 4.9. Stomatal Aperture Measurements

Abaxial epidermal strips were rapidly prepared from the control and flg22-treated, as well as distal, leaves of intact WT or *Nr* plants, then immediately observed under a microscope (Nikon Eclipse TS-100, Nikon Instruments, Tokyo, Japan) as earlier described by Melotto et al. [125]. The width of stomatal pores was measured using the Image-Pro Plus 5.1 software (Media Cybernetics, Inc., Rockville, MD, USA). Leaves of three different plants were used for sampling in the case of each treatment. A total of 30–40 stomata of three different plants was recorded and the measurements were repeated 3 times using new plant generations.

### 4.10. Statistical Analysis

Experiments were repeated at least two times. The results are expressed as means ± S.E. The effects of flg22 treatments and the differences among the times of day were analysed by two-way ANOVA, supplemented with post hoc pairwise comparisons using Duncan’s multiple range test. All statistical analyses were performed using the SigmaPlot 11 software (Systat Software Inc., San Jose, CA, USA). Mean values denoted with different letters and differed significantly at *p* < 0.05.

## 5. Conclusions

Understanding the mechanism that can regulate plant defence mechanisms at the molecular and physiological or whole plant levels is an important problem in current plant biology, as well as in agriculture. It can be concluded that flg22-induced rapid local and systemic defence responses in the first hour are dependent on the day/night-time of the bacterial elicitor treatment. In addition, local and systemic responses of intact tomato plants are mediated by ET in the light period. ET influences H_2_O_2_ and NO production, as well as JA and SA levels and signalling based on the expression of *PR1* and *DEF* locally and superoxide production systemically, contributing to the initiation of stomatal closure in WT plants in the light phase of the day. Enhanced *ERF1* and *DEF* expression was also observable in systemic leaves of WT plants in the light and was dependent on the active ET signalling. This research provides new results to understand the local and systemic role of ET in the biotic stress responses of intact plants in the context of other defence-related phytohormones, such as SA and JA. In addition, our results demonstrate the differences in plant defence responses in the daytime and at night. Based on this knowledge, the potential modification of phytohormone levels, such as the ET production at night, when most of the plant pathogens are more active, as compared to daytime, may be used to increase the yield and stress tolerance under biotic stress conditions.

## Data Availability

Tomato genes originated from the Sol Genomics Network (SGN; http://solgenomics.net/, accessed on 31 January 2021) database. The authors affirm that all data necessary for confirming the conclusions of the article are present within the article and figures.
